# Mathematical Modeling of the Effectiveness of Facemasks in Reducing the Spread of Novel Influenza A (H1N1)

**DOI:** 10.1371/journal.pone.0009018

**Published:** 2010-02-10

**Authors:** Samantha M. Tracht, Sara Y. Del Valle, James M. Hyman

**Affiliations:** 1 Energy and Infrastructure Analysis Group, Decisions Applications Division, Los Alamos National Laboratory, Los Alamos, New Mexico, United States of America; 2 Department of Mathematics, Computer Science, and Physics, Capital University, Columbus, Ohio, United States of America; 3 Mathematical Modeling and Analysis Group, Theoretical Division, Los Alamos National Laboratory, Los Alamos, New Mexico, United States of America; University of Sydney, Australia

## Abstract

On June 11, 2009, the World Health Organization declared the outbreak of novel influenza A (H1N1) a pandemic. With limited supplies of antivirals and vaccines, countries and individuals are looking at other ways to reduce the spread of pandemic (H1N1) 2009, particularly options that are cost effective and relatively easy to implement. Recent experiences with the 2003 SARS and 2009 H1N1 epidemics have shown that people are willing to wear facemasks to protect themselves against infection; however, little research has been done to quantify the impact of using facemasks in reducing the spread of disease. We construct and analyze a mathematical model for a population in which some people wear facemasks during the pandemic and quantify impact of these masks on the spread of influenza. To estimate the parameter values used for the effectiveness of facemasks, we used available data from studies on N95 respirators and surgical facemasks. The results show that if N95 respirators are only 20% effective in reducing susceptibility and infectivity, only 10% of the population would have to wear them to reduce the number of influenza A (H1N1) cases by 20%. We can conclude from our model that, if worn properly, facemasks are an effective intervention strategy in reducing the spread of pandemic (H1N1) 2009.

## Introduction

Novel influenza A (H1N1) (hereafter referred to as pandemic (H1N1) 2009 in keeping with the World Health Organization (WHO) nomenclature) is a new flu virus of swine, avian, and human origin that was first identified in mid-April 2009 in Mexico and the United States [1]. The virus soon spread to the rest of the world and on June 11, 2009 the WHO declared novel influenza A (H1N1) a pandemic. The virus continues to spread, with most countries reporting cases of pandemic (H1N1) 2009 [1]. Even though the WHO's declaration of a phase six pandemic alert level does not explicitly refer to the severity of the disease, as many people contracting the virus recover without medical treatment, the number of deaths continues to rise [1]. The rapid spread of influenza, due to its short incubation period and lack of strain-specific vaccine, pose a challenge to the implementation of effective mitigation strategies during the expected reemergence of pandemic (H1N1) 2009 in the fall/winter flu season. Every year approximately 36,000 people die from seasonal influenza or flu-related causes in the U.S. [2]. However, the number of casualties may increase with a new and more virulent strains of influenza, such as the pandemic (H1N1) 2009.

The emergence of an unexpected or new strain of influenza means there are no prepared vaccines and the existing antivirals may be ineffective in combating the spread of infection. Vaccination is typically the first line of defense against influenza viruses [3]. The entire vaccine production process takes at least six months to complete [4] and although a pandemic (H1N1) 2009 vaccine became available in the U.S. in October 2009, there are severe shortages in the amount of vaccines available. Another concern is that the currently circulating H1N1 strain could mutate, making the vaccine ineffective or less effective.

In the recent pandemic (H1N1) 2009 outbreak, non-pharmaceutical interventions such as school closings and thermal screenings at airports were implemented to slow the spread of disease [5], [6]. Other common non-pharmecuetical interventions include quarantine, isolation, travel restrictions, closing of public places, fear-based self quarantine, and cancellation of events. These interventions all have economic costs to individuals and society related to lost work, increased school absenteeism, and decreased business revenues.

Another non-pharmaceutical option is the use of facemasks. In the 2003 SARS outbreak many individuals used facemasks to reduce their chances of contracting infection. In Hong Kong 76% of the residents reported using masks during the 2003 SARS epidemic [7]. Even though individuals have taken upon themselves to wear facemasks during disease outbreaks, little research has been done to quantify the impact of the use of facemasks during an epidemic. Mathematical models of the spread of infectious disease can be useful in assessing the impact of facemasks on reducing the spread of a disease, specifically pandemic (H1N1) 2009.

### Mask Studies

Pandemic (H1N1) 2009 spreads through person-to-person contact, airborne particles, coughing and sneezing, and by fomites [1], therefore, the use of facemasks is a logical line of defense. The Centers for Disease Control and Prevention (CDC) have interim recommendations on the use of facemasks and respirators for the current pandemic (H1N1) 2009 virus. The CDC defines the term *facemask* as a disposable mask cleared by the U.S. Food and Drug Administration (FDA) for use as a medical device, such as surgical masks. Surgical masks are designed to help stop droplets from being spread by the person wearing the mask, not to protect against breathing in very small particle aerosols that may contain viruses [8]. We will use of the term ‘respirator’ for an N95 or higher filtering facepiece respirator certified by the CDC/National Institute for Occupational Safety and Health (NIOSH); a respirator is designed to protect the person wearing the mask against breathing in very small particles that may contain viruses [8]. The CDC states that the effectiveness of the use of facemasks and respirators in various settings is unknown and do not generally recommend the use of facemasks or respirators in home or community settings nor in non-medical occupational settings [8]. In certain circumstances the CDC recommends the use of masks for individuals who are at high risk of infection and cannot avoid situations with potential exposure to the disease [8].

There have been a handful of studies that have analyzed the effectiveness of facemasks against nanoparticles in the size range of viruses using manikin-based protocol in which the masks were sealed on the manikin's face so that no leakage would occur [9]–[11]. All three studies show similar results in penetration percentage for the N95 respirator. The high fit N95 respirator had penetration percentages from about 0.5% to 2.5% at 30 l/min and from about 0.5% to 5% at 85 l/min [9]–[11]. The low fit N95 respirator had penetration percentages from about 1.5% to 3.5% at 30 l/min and from about 1.5% to 6% at 85 l/min [9]–[11]. The surgical masks tested in Balazy et al.'s [10] study show a much greater penetration percentage. At 30 l/min one model of surgical mask (SM1) allowed 20–80% of particles to penetrate the mask, while another model (SM2) allowed 2–15% [10]. At 85 l/min SM1 allowed penetration of 30–85% of particles while SM2 allowed 5–21% [10]. The N95 respirator in a sealed manikin test seems to be fairly effective against nanoparticles, almost holding up to its 95% certification. The surgical masks are not as effective, allowing a much greater percentage of particles to pass through to the wearer even when sealed tightly to a manikin.

Unfortunately, this type of testing does not provide an accurate estimate of the level of protection for everyday use of a mask by a person. While these studies provide data on the actual protection of masks against nanoparticles in a perfect setting, it does not take into consideration that a mask will not be completely sealed on an individual nor will it fit perfectly. Furthermore, one must consider that an individual will not always be wearing the mask, for example, a mask will be taken off to eat and sleep, or possibly because it becomes uncomfortable to wear.

Lee et al. [12] performed a study on N95 respirators and surgical masks using human subjects. The challenge aerosol used was NaCl, with particles in the size range of bacteria and viruses (.04–1.3
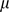
m). They tested four models of N95 respirators: 1) high protection level, 2) medium protection level, 3) exhalation valve, and 4) exhalation without valve and three models of surgical masks: 1) high protection level, 2) medium protection level, and 3) low protection level. The results from the study showed that the lowest protection offered from N95 respirators is when particles are in the size range of 0.08–0.2
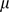
m and for surgical masks when particles are in the size range of 0.04–0.32
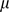
m. The size range of influenza virus is in the range of 0.08–0.12
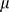
m, which falls into both masks most penetrating particle size range. The N95 respirator was found to be 21.5% effective and the surgical mask was 2.4% effective in protecting against nanoparticles. The N95 respirator provides approximately nine times greater protection than a surgical mask and is clearly a better option in protecting against infection.

A University of Michigan School of Public Health study led by Dr. Allison Aiello [13] is evaluating the effectiveness of hand-washing and facemasks in preventing influenza from spreading. The study, called M-FLU, conducted a randomized cluster intervention trial among students living in dorm housing. The students were randomly separated into two intervention groups, one wearing masks and practicing hand hygiene, one just wearing masks, and also in a control group. The study was carried out over the 2006–2007 influenza season, which was a mild season. The study found that facemasks and hand hygiene were correlated with a 35–51% reduction in influenza-like illness [13].

There are many factors that influence people's willingness to wear a mask. In a study by Tang and Wong [14] a total of 1,329 adult Chinese residing in Hong Kong were surveyed on their use of facemasks during the 2003 SARS epidemic. Overall 61.2% of the respondents reported the consistent use of facemasks to prevent contracting the disease. The study found that women in the age group 50–59 and married respondents were more likely to wear facemasks, suggesting that the aesthetics of wearing a facemask may be a concern. Also, the study found that individuals who had a university education or earned more than US$5,000 per month were more likely to wear a mask. Tang and Wong also showed that perceived susceptibility, cues to action, and perceived benefits, were significant predictors in whether or not an individual consistently wore a mask.

## Methods

**Figure 1:**
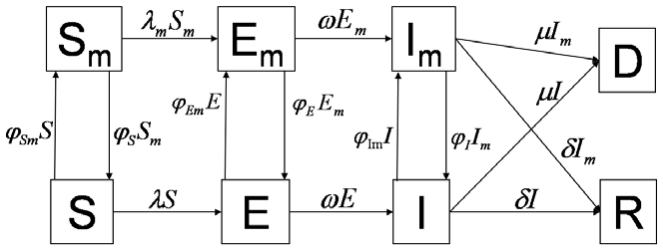
Schematic relationship between mask wearing individuals and non-mask wearing individuals for pandemic (H1N1) 2009. The arrows that connect the boxed groups represent the movement of individuals from one group to an adjacent one. Non-mask wearing susceptible individuals (S) can either become exposed (E) or susceptible wearing a mask 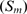. Non-mask wearing exposed individuals (E) can either become infectious non-mask wearing (I) or mask wearing exposed (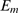). Non-mask wearing infectious individuals (I) can either recover (R), die (D), or become infectious wearing a mask (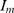). Mask wearing susceptible individual (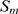) can either become an exposed mask wearer (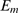) or a non-mask wearing susceptible (S). Mask wearing exposed individuals (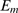) can either become an infectious mask wearer (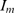) or a non-mask wearing exposed individual (E). A mask wearing infectious individual (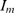) can either recover (R), die (D), or stop wearing the mask while they are still infectious (I).

Following the approached developed in [15], the population is divided into two subgroups: a mask wearing group (subscript *m*) and a non-mask wearing group. People move back and forth between the mask and non-mask groups based on the number of individuals infected with pandemic (H1N1) 2009. Individuals in each activity group are characterized by their epidemiological status: susceptible, denoted by S and 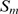, exposed, denoted by E and 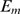 (i.e., people who are infected but not yet fully contagious), and infectious individuals, I and 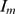. Definitions of the eight epidemiological classes are summarized in Table 1 and the transfers are shown diagrammatically in Figure 1. Because we are evaluating the effectiveness of masks in a single influenza period, we use a closed system with no migration in or out, and births and natural deaths are not included in the model.

**Table 1:** State Variables for the Model.

Variable	Definition
S	Number of Susceptible Individuals Not Wearing a Mask
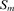	Number of Susceptible Individuals Wearing a Mask
E	Number of Exposed Individuals Not Wearing a Mask
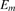	Number of Exposed Individuals Wearing a Mask
I	Number of Infected Individuals Not Wearing a Mask
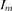	Number of Infected Individuals Wearing a Mask
R	Number of Recovered Individuals
D	Number of Dead Individuals

As seen in Figure 1, the transfer rates of people from the exposed classes, E and 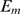, to the infectious classes, I and 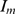, are 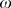
E and 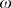 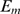. Infectious individuals can move to group D, at rate 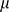
I and 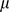 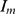, when they die from infection or to group R, at rate 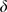
I and 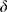 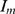, upon recovery. The mean times in the infectious classes, I and 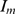, are 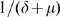. Hence, the infectious fraction 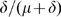 recovers and the infectious fraction 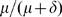 dies as a consequence of this disease.

We assume that there is homogeneous mixing between groups and that contact activity levels remain normal throughout the epidemic. We define 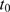 as the beginning of the epidemic. Movement of individuals between mask and non-mask groups depends upon the number of pandemic (H1N1) 2009 cases in the population. A specified percentage of the population starts wearing masks as the number of infected people increases.

We define 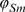
S, 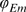
E, and 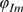
I to be the transfer rates from the S, E, and I classes to the 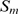, 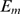, and 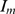 classes, respectively, similarly 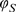 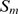, 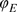 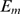, and 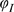 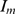 are the transfer rates from the 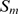, 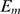, and 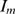 classes to the S, E, and I classes, respectively.

The rate coefficients are modeled by step-functions of the number of infectious individuals:
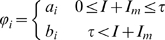
(1)for i = S, E, I, 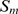, 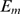, and 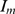. Here the parameters *a* and *b* are positive constants that determine the rate of movement and 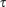 is the number of pandemic (H1N1) 2009 cases that determines when masks are implemented. For i = S, E, and I, 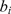 is set at 0.1 or 10% of the population.

Using the transfer diagrams in Figure 1 we obtain the following system of differential equations:
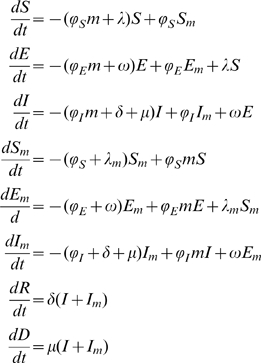
(2)


Here 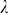 (non-mask group) and 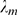 (mask group) are the forces of infection and 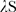
S and 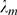 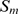 are the transfer rates from the susceptible classes, S and 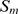, to the exposed classes, E and 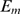. The infection rates, 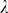 and 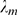, incorporate the probability of transmission per contact, 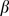, the reduced infectiousness due to incubation, 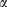, the reduced number of contacts because of symptomatic infection, 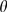, and 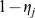, (*j* = *s* or *i*), which accounts for the effectiveness of the mask in reducing either susceptibility (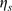) or infectivity (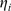). The transmissibility, 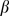, is defined as the susceptibility of the population multiplied by the infectivity of the disease multiplied by the average number of contacts an individual has per day. The definitions of the parameters are summarized in Table 2. The forces of infection for the non-mask group and mask group are shown by:
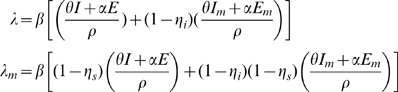
(3)where 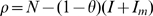 and N is the total population 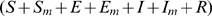. In the force of infection, (1-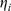) multiplies the 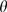 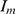/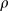 and 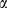 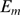/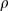 infectious fractions because individuals in the 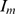 and 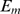 classes are wearing masks. Also, (1-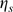) multiplies the infectious fractions in 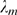 because individuals in the susceptible class (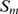) are wearing masks. These forces of infection and appropriate initial conditions complete our model formulation.

**Table 2:** Parameter Definitions and Values.

Parameter	Description	Units	Baseline	Range	Reference
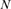	Total Population	People	1 million	0–300 million	See text
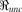	Effective Reproduction Number (uncontrolled)	1	1.83	0–2	[16], [24]–[26], [33]
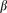	Transmission Rate	1	0.23	0–1	[27]–[29]
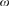	Incubation Relative Rate	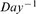	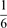	0–1	[18]
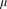	Death Relative Rate	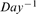	0.001	0–1	[25], [26], [34]–[36]
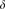	Recovery Relative Rate	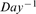	0.2	0–1	[20]
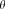	Reduced Number of Contacts Due To Illness	1	1	0–1	[28]
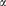	Reduced Infectiousness Due to Incubation	1	0.5	0–1	[19], [31]
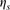	(N95) Decrease in Susceptibly because of Mask	1	0.20	0–1	[12]
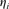	(N95) Decrease in Infectivity because of Mask	1	0.5	0–1	[12]
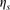	(SM) Decrease in Susceptibly because of Mask	1	0.02	0–1	[12]
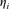	(SM) Decrease in Infectivity because of Mask	1	0.05	0–1	[12]
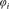	Movement Rate Between Classes	1	See text	0–1	See text
	i = S, Sm, E, Em, I, Im				
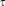	Number of Infecteds at which Mask are Implemented	People	100	100–10000	See text
I/N	Initially Infected Fraction of the Population	1	0.00001	0–1	See text

### The Effective Reproduction Number 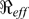


The effective reproduction number, 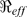, is the average number of secondary cases produced by a typical infectious individual during the infectious period [16], [17]. The effectiveness of intervention strategies are often measured by their ability to reduce the spread of a disease in a given population. In an epidemic model the magnitude of the effective reproduction number, 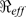, determines whether or not an epidemic occurs and its severity [15]. When 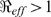, the number of infections grow and an epidemic occurs, however when 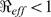, the number of infections does not increase and there is no epidemic outbreak [15].

Without any interventions the model has an initial effective reproduction number (uncontrolled) 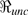 given by:
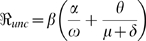
(4)


This 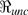 is the product of the average number of people infected per unit time 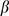 and the weighted sum of the average infectious period 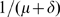 plus the average incubation period 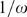.

The ‘next-generation operator’ approach [17] is used to find an expression for the effective reproduction number (controlled) 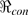 for our epidemic model when masks are used as an intervention strategy. The computation is done by linearizing the system of equations (2) around the disease-free equilibrium (DFE). The DFE has E, 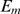, I, and 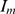 equal to zero with 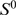, 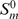, and 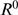 positive. Since there is no immunity from previous infection or vaccination 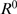 is also equal to zero. The resulting four-dimensional linearized system is of the form 
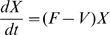
, where
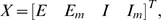
(5)

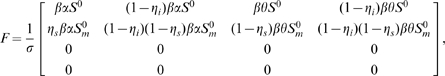


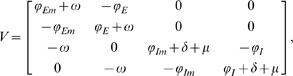



The effective reproduction number 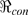 is the largest eigenvalue of the matrix 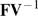 [17]. Hence 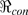 is the only non-zero eigenvalue of the matrix 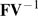 and is given by the expression:
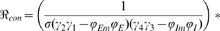
(6)

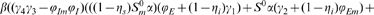


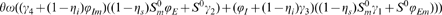
where 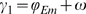, 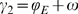, 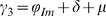, 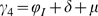, and 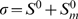.

We use equations 4 and 7 to define the effective reproduction number for the model as:
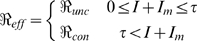
where 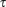 is the threshold number of infected individuals at which masks start to be used.

### Estimation of Parameter Values

The epidemiology of pandemic (H1N1) 2009 is not accurately known since it continues to spread across the world. The parameter values shown in Table 2 were chosen based on the best available data. The incubation period for pandemic (H1N1) 2009 has been reported to be 2–10 days with a mean of 6 days [18]. The mean time in the exposed classes E and 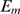 corresponding to the incubation period has been assumed to be 6 days, making the transfer rate to the infectious classes, I and 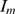, constant at 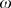 = 1/6.

The infectious period is believed to be between four and seven days, with an average of five days [19], [20]. Thus, the baseline value for the recovery rate is constant at 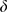 = 1/5. The fatality rate of the pandemic (H1N1) 2009 is thought to be in the range of 0.3%–1.5%, with a mean of 0.46% [21]–[23]. The case fatality rate for our model is 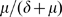, setting this equal to 0.0046 results in 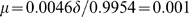.

The current estimates on the transmission of pandemic (H1N1) 2009 are that one infected person may typically infects one to two people [24]–[26]. The transmissibility, 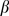, is the product of the susceptibility of the population, the infectivity of the disease, and the number of contacts an individual has in a day [27], [28]. The susceptibility of the population is set to one, as it is believed few people are immune to pandemic (H1N1) 2009, and the number of contacts an individual has per day is assumed to be 16 [29]. The infectivity is found by 
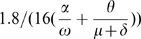
, so that 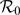 = 1.8 in a completely susceptible population and the infectivity is .0141. So 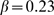 gives the transmission rate, the fraction of contacts per day that is sufficient for the transmission of pandemic (H1N1) 2009.

The baseline population size N for the model is set at one million people and all are initially in the susceptible class S. The initial infected fraction, I/N, is set at 0.00001 so that when N = 1000000, I = 10. The model scales linearly so that the initial population size N and the initial number of infected individuals I are both scaled up or down by the same factor. We assume that individuals will start wearing masks after 100 people are infected, once there is enough number of cases in a community to convince people to start wearing masks. We analyzed the impact of masks when 10%, 25%, and 50% of the population wear them.

Using the studies published on the effectiveness of masks we determined the baseline values for the effectiveness of N95 respirators to be 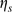 = 0.2 and 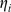 = 0.5 and for the surgical masks 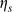 = 0.02 and 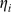 = 0.05 [12]. The effectiveness of masks in decreasing the infectivity of a sick individual is greater because the mask contains the virus particles, preventing them from becoming airborne, and therefore preventing the contamination of surrounding surfaces as well as people [30].

Although it is possible that some sick individuals may change their behavior due to the symptoms [15], we assume that sick individuals will not change their behavior and continue to have the same number of daily contacts as a healthy individual. Therefore, we set the baseline value for the reduced number of contacts due to illness 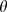 at 1, as people usually do not greatly alter their daily behavior during the incubation period. Individuals in the exposed classes, E and 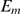, are thought to be 50% less infectious due to incubation than those in the infected classes, I and 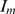, so we set 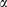 = 0.5 [19], [31].

## Results

We analyzed two scenarios: one in which the N95 respirator is worn and one in which surgical masks are worn; for both types of masks we considered three different variations in mask effectiveness. Each case is evaluated with 10%, 25%, and 50% of susceptible and exposed individuals wearing masks, while in each case the fraction of infectious individuals wearing masks is slightly larger. When 10%, 25%, and 50% of susceptible and exposed individuals are wearing masks the fraction of infectious individuals wearing masks is 30%, 40%, and 50%, respectively. All simulations assume that in a population of one million there are initially 10 infected individuals reported and everyone else is susceptible. Mask start being used when there have been 100 reported cases of pandemic (H1N1) 2009.

**Figure 2:**
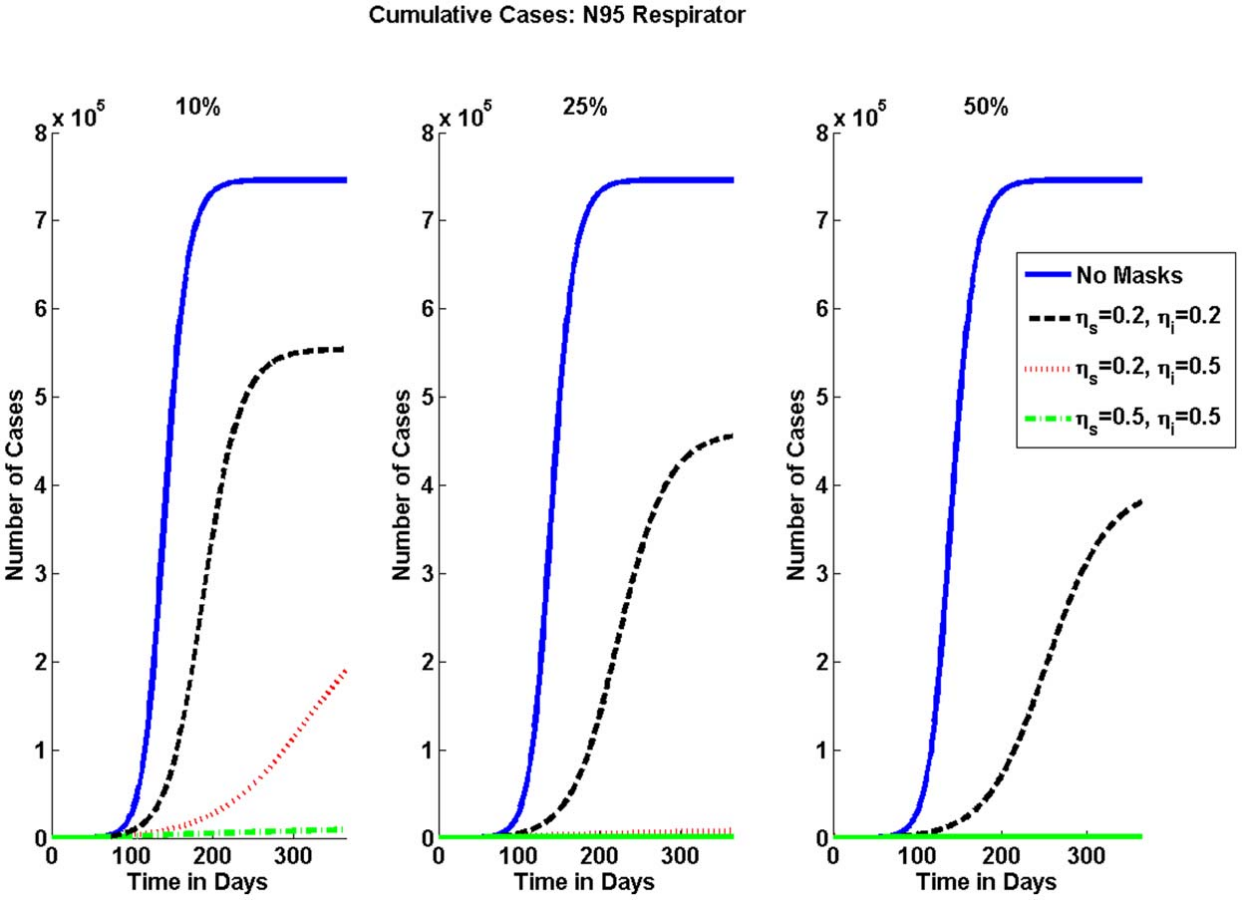
Cumulative Number of Cases for N95 Respirator. Without any interventions the number of cumulative cases is shown by the solid blue line. As expected when the mask is more effective or more people wear a masks, then the number of cumulative cases decreases. Note how effective the N95 is when only 10% of the population wears a respirator.

**Figure 3:**
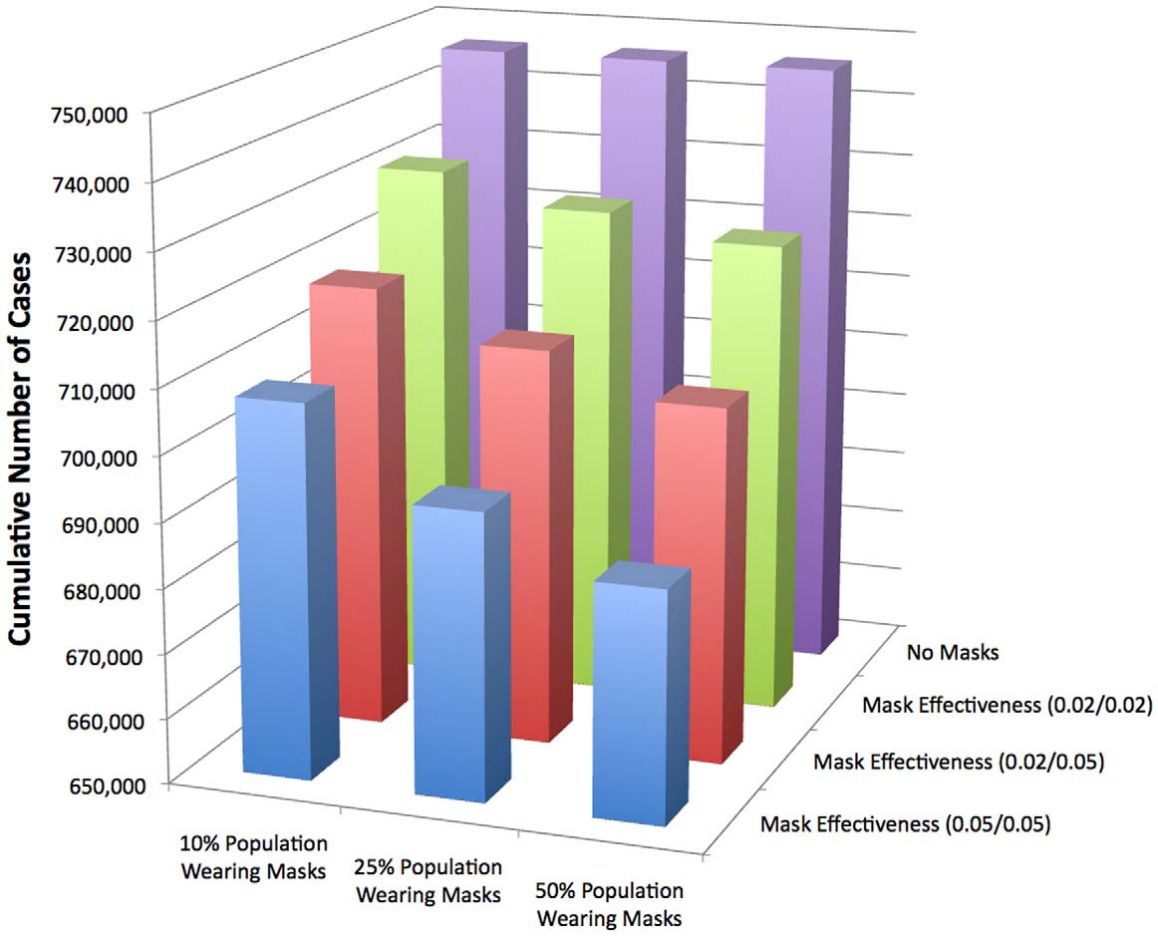
Cumulative Number of Cases for Surgical Masks. The same pattern that was seen in Figure 2 with respirators is also seen here: as the masks effectiveness is higher the number of cumulative cases decreases and the number of cases also decreases if a higher percentage of people wear masks. However, the difference in the number of cumulative cases is not nearly as large when surgical masks are worn; this is due to their lower effectiveness.

The numerical results for the percentage of pandemic (H1N1) 2009 cases are shown in Table 3 for the N95 respirator and in Table 4 for surgical masks. The effective reproduction numbers for each case are shown in Table 5 for N95 respirators and in Table 6 for surgical masks. The cumulative number of pandemic (H1N1) 2009 cases can be seen graphically for the varying mask effectiveness and the different fractions of individuals wearing masks in Figure 2 and in Figure 3 for N95 respirators and surgical masks, respectively.

**Table 3:** Percentage of the Number of Cumulative Cases in a Population of 1 Million: N95 Respirators.

N95 Respirator Effectiveness		Percentage of	Population Wearing	N95 Respirators
Susceptible ( 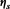 )	Infectious ( 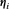 )	10%	25%	50%
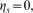	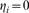	74.61	74.61	74.61
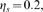	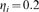	55.39	45.56	38.09
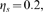	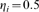	11.92	0.81	0.30
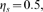	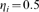	0.94	0.18	0.10

Percentage of the number of cumulative cases in a population of one million for varying percentages of population wearing N95 respirators and varying mask effectiveness for susceptibles (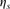) and infectious (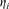). Notice that as a higher percentage of people wear masks there is a lower percentage of cumulative cases. Also, as mask effectiveness increases the percentage of cases goes down.

**Table 4:** Percentage of the Number of Cumulative Cases in a Population of 1 Million: Surgical Masks.

Surgical Mask Effectiveness		Percentage of	Population Wearing	Surgical Masks
Susceptible ( 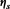 )	Infectious ( 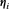 )	10%	25%	50%
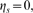	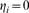	74.61	74.61	74.61
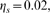	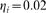	73.13	72.68	72.34
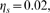	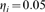	71.85	71.12	70.49
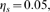	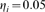	70.75	69.40	68.55

Percentage of the number of cumulative cases in a population of one million for varying percentages of population wearing surgical masks and varying mask effectiveness for susceptibles (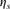) and infectious (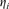). Notice that as a higher percentage of people wear masks there is a lower percentage of cumulative cases. Also, as mask effectiveness increases the percentage of cases goes down. Note surgical masks do not decrease the percentage of the number of cases as greatly as N95 respirators.

**Table 5:** Effective Reproduction Number, 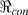: N95 Respirator.

Respirator Effectiveness		Effective Reproduction Number, 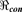		
Susceptible ( 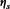 )	Infectious ( 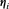 )	10%	25%	50%
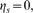	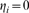	1.83	1.83	1.83
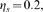	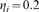	1.66	1.6	1.56
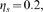	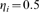	1.4	1.26	1.16
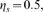	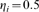	1.4	1.26	1.16

Effective Reproduction Number 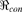 for N95 respirators. Notice that 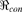 decreases as a higher percentage of people wear masks as well as when masks are more effective. 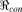 is greatly reduced when 50% of the population wears masks and masks are 50% effective.

**Table 6:** Effective Reproduction Number, 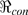: Surgical Masks.

Mask Effectiveness		Effective Reproduction Number, 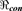		
Susceptible ( 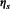 )	Infectious ( 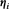 )	10%	25%	50%
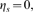	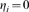	1.83	1.83	1.83
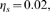	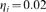	1.81	1.81	1.8
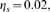	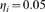	1.79	1.77	1.77
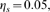	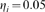	1.79	1.77	1.77

Effective Reproduction Number, 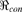, for surgical masks. Notice that 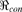 decreases as a higher percentage of people wear masks as well as when masks are more effective. However, 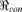 is not greatly reduced even when 50% of the population wears masks and masks are 50% effective.

Table 3 and Table 4 show that when masks are not used, then the total percentage of the population who will be infected is 74.61% in a population of 1 million people. With the implementation of N95 respirators Table 3 exhibits a reduction in the cumulative number of cases of almost 200,000, or a 19% decrease, when 10% of the population wears masks and they are 20% effective. Table 5 shows the implementation of the N95 Respirators' impact on the effective reproduction number 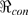; it is reduced from 1.83 to 1.66 when masks are 20% effective in reducing both susceptibility and infectivity and 10% of the population is wearing masks. When effectiveness is increased to 50% 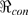 is reduced even further to 1.4. As the fraction of the population wearing N95 respirators increases, 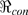 is reduced even further, and at the lowest is 1.16. Table 4 shows that surgical masks do not have as large of an impact in reducing the cumulative number of cases as does the N95 respirator. Table 6 displays the effective reproduction number 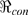 when surgical masks are implemented. The lowest value surgical masks reduce 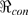 to is 1.77.

In Figure 2 the effectiveness of the N95 respirator in reducing the spread of pandemic (H1N1) 2009 is significant. As the percentage of the population wearing masks increases the number of cumulative cases decreases and when the mask effectiveness is greater, the number of cases is also greatly reduced. The impact of surgical masks is not as large as seen graphically in Figure 3, the reduction in the cumulative number of cases is relatively small compared to that of the N95 respirator. If mask effectiveness is 5% and 50% of the population wears surgical masks the reduction in the number of cumulative cases is 6%.

### Sensitivity Analysis

Even though the parameter values were estimated from epidemiological data, there is still some uncertainty in their values. Since pandemic (H1N1) 2009 is a new virus, there is a wide range of estimated values for the parameters. In our model we chose the averages for our baseline parameters, here we look at a range of parameters and how changing a specific one effects the outcome of the model. This sensitivity analysis examines the effects of changes in the reproduction number (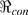), mask effectiveness (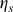 and 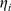), index cases (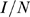), fraction of population wearing masks (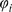), number of initially infected at which masks are implemented (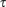), as well as the effect of which epidemiological group wears masks (S or I). Unless otherwise stated the other parameters are fixed at their baselines values found in Table 2.

#### Effective reproduction number

**Figure 4:**
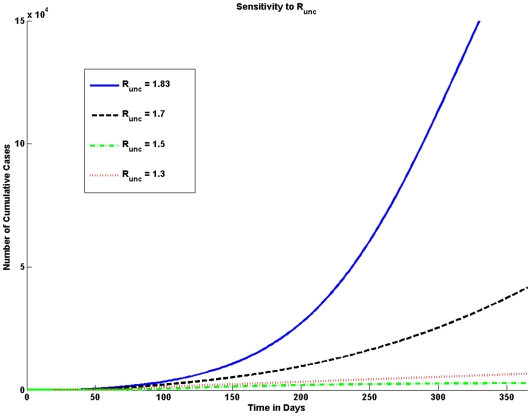
Sensitivity to 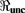. The number of cumulative cases is very sensitive to the value of the uncontrolled effective reproduction number (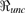 ). Higher values of 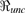 result in a larger number of cumulative cases. A large difference in the number of cases is seen when the 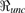 is equal to 1.83 and when 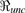 is equal to 1.7; for such a slight difference in 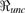 the difference in the number of cases is quite large.

The effective reproduction number 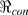 determines the average number of secondary cases resulting from one typical infectious individual during the infectious period without the implementation of facemasks. Since there is a delay in the implementation of facemasks the initial growth of the epidemic is affected by 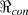. The estimates of 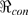 for pandemic (H1N1) 2009 vary widely, the common range is assumed to be between 1.2 and 2.2. As the value of 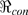 increases the number of pandemic (H1N1) 2009 cases increases significantly as shown graphically in Figure 4.

#### Mask effectiveness

The effectiveness of the mask greatly affects the number of cumulative cases. The higher the effectiveness the fewer number of cases (shown in the Results section). The effectiveness of the masks not only depends upon the type of mask and quality but also proper usage.

#### Index cases

**Figure 5:**
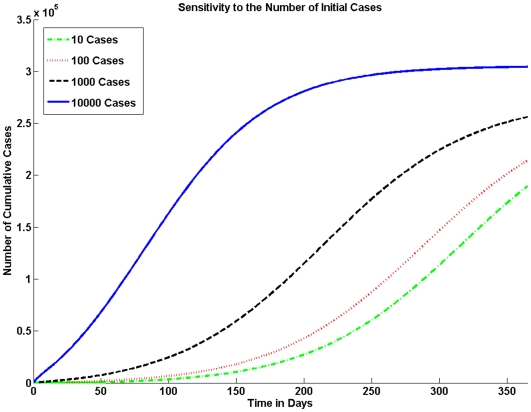
Sensitivity to the Number of Initial Cases. The model is sensitive to the number of index cases. In a population of one million if the number of index cases is 10 there are significantly fewer cases than if the number of index cases is 1000 or 10,000.

The number of initially infected individuals can have a major impact on the size of the epidemic. In Figure 5 we vary the number of initially infected individuals in the population.

#### Fraction of population wearing masks

**Figure 6:**
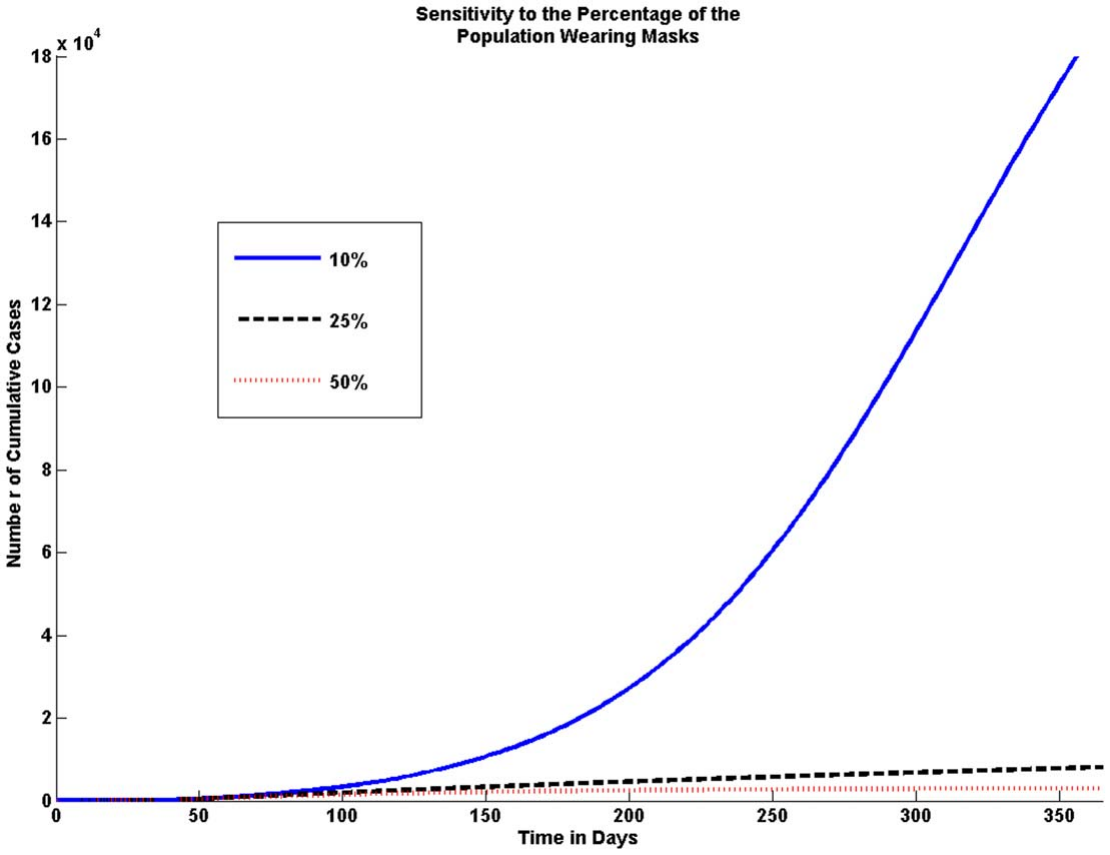
Sensitivity to the Percentage of the Population Wearing Masks. The fraction of the population wearing masks greatly affects the number of cases. Even if only 10% of the population wears masks the number of cumulative cases is significantly reduced; however, the graph shows that the number of cases is drastically reduced if 25% of people wear masks.

We consider variations in the percentage of the population that wears masks. We look at the effect of 10%, 25% and 50% of the population wearing masks. The model shows that the higher the percentage of the population wearing masks the fewer the number of cumulative cases, this is shown in Figure 6.

#### Implementation of masks

**Figure 7:**
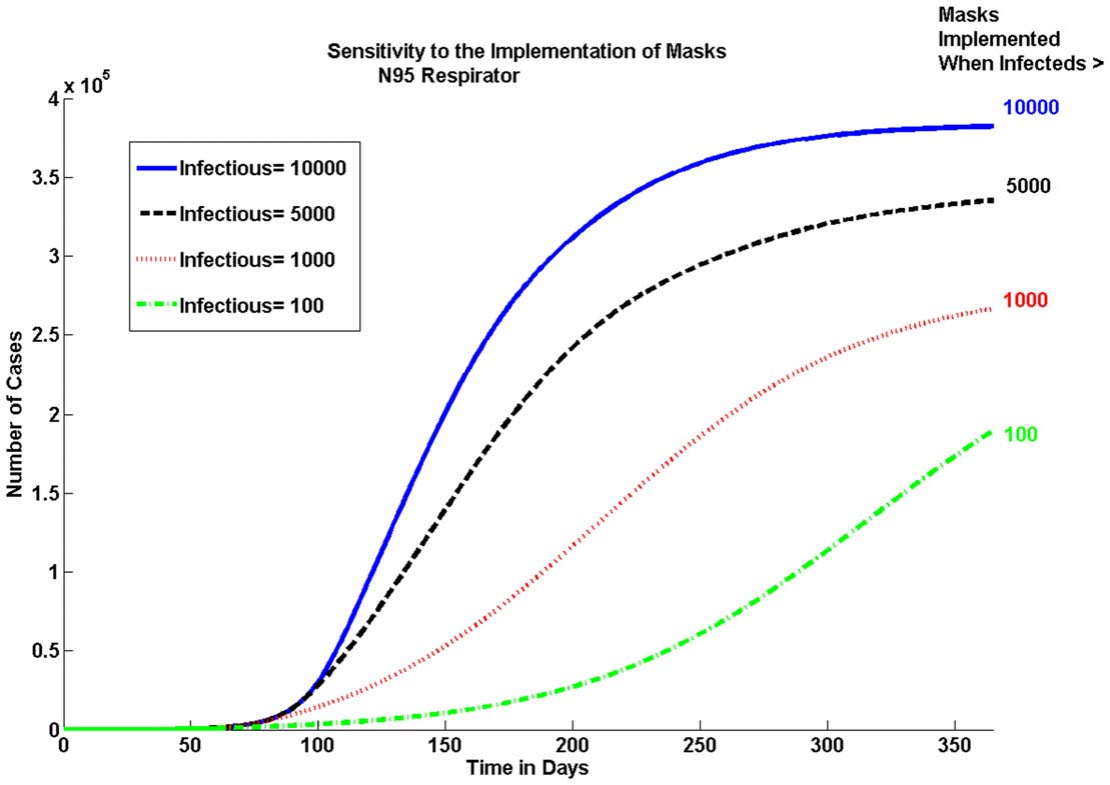
Sensitivity to When Masks Are Implemented. Masks should be implemented as soon as possible. There is a large difference in the number of cases when masks are implemented at 100 infectious individuals versus waiting until there are 1000.

The epidemic is sensitive to the delay in the implementation of masks as seen in Figure 7. We look at the cumulative number of pandemic (H1N1) 2009 cases for the N95 respirator when 10% of the population is wearing masks. Figure 7 shows that the earlier masks are implemented, the bigger the reduction in the cumulative number of cases.

#### Who wears masks

**Figure 8:**
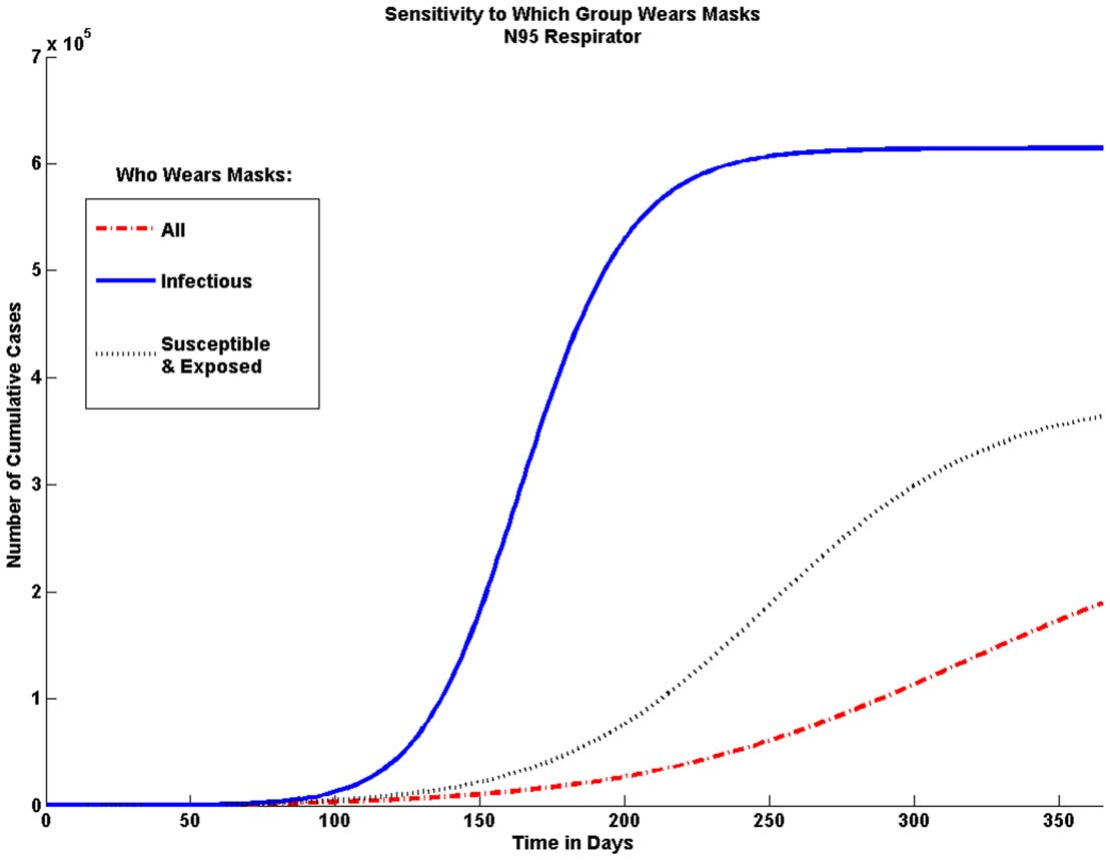
Sensitivity to Who Wears Masks. In order to achieve the greatest possible reduction in the cumulative number of cases both infectious individuals and susceptible and exposed individuals should wear masks. If only infectious individuals wear masks the number of cases is not significantly reduced.

The model is sensitive to who wears masks. Here we look at the effect if only infected individuals wear masks and if only susceptible and exposed individuals would wear masks. Figure 8 shows that it is important for both infected, as well as susceptible and exposed individuals, to wear masks.

## Discussion

The standard mitigation strategies used for influenza viruses are vaccines and antivirals. However, in the case of a novel virus these may not be readily available and other mitigation strategies will be needed. As seen during the 2003 SARS outbreak and the current pandemic (H1N1) 2009 people are willing to wear facemasks to reduce the spread of disease. We used a mathematical model to examine the possible impact of N95 respirators and surgical masks on reducing the spread of pandemic (H1N1) 2009. When modeled with a low mask effectiveness and a small fraction of the population wearing masks, the implementation of facemasks still has a relatively large impact on the size of the pandemic (H1N1) 2009.

The numerical simulation results in the results section show that without any interventions, we predict that a large percentage of the population will be infected with pandemic (H1N1) 2009 influenza strain. This result is not surprising as the population is 100% susceptible and the effective reproduction number 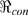 is 1.83, which is higher than that of typical seasonal influenza. In reality, the 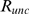 may be lower due to heterogeneous mixing patterns, pre-existing immunity, and other interventions in place. With 10% of the population wearing N95 respirators with effectiveness at 20% in reducing both susceptibility and infectivity there is a 19% reduction in the cumulative number of cases. With the same mask effectiveness but 25% of the population wearing N95 respirators, the total number of pandemic (H1N1) 2009 cases is reduced by almost 30% and with 50% of the population wearing masks, it results in over a 36% reduction in the number of cases.

The effectiveness of surgical masks is low, therefore the impact of wearing them during an epidemic is not significant. Even at 50% effectiveness in reducing both susceptibility and infectivity and with 50% of the population wearing surgical masks only a 6% reduction in the number of cumulative cases is seen.

The sooner an epidemic is recognized and masks are implemented, the bigger the reduction in the number of cases will be. As seen in the results section the epidemic is sensitive to the delay in implementing masks. The difference in the total number of pandemic (H1N1) 2009 cases when masks are implemented at 100 infected individuals and 1,000 infected individuals is over 7%.

The implementation of neither N95 respirators nor surgical masks lowered the effective reproduction number 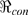 below one. However, N95 respirators greatly decreased 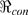, in some scenarios very close to one. While facemasks will not stop the pandemic (H1N1) 2009, they could greatly reduce its severity and allow for more time to develop effective vaccines and antivirals.

There are currently more trials being conducted on the effectiveness of surgical masks and N95 respirators [32], which will allow us to refine the assumptions made in the model. However, it must be noted that in order for masks to be effective they must be: (1) available, (2) affordable, (3) worn properly, (4) replaced or sanitized daily, and (5) N95 respirators should be fit-tested. Only 10% of the population would have to wear masks in order to reduce the percentage of cases by 20%. Facemasks are inexpensive, relatively easy to implement, and would not cause a large economic burden to society. Masks are a powerful tool and can be used by countries with limited supplies of antiviral drugs and vaccines. In addition, economically feasible preventative global mitigations will benefit the world as a whole. We can conclude from our model that N95 respirators if worn properly are an effective intervention strategy in reducing the spread of the pandemic (H1N1) 2009.
